# Balloon-occluded retrograde transvenous obliteration of colonic varices: a case report

**DOI:** 10.1186/s42155-020-00108-3

**Published:** 2020-03-16

**Authors:** Chantal Liu, Sivasubramanian Srinivasan, Suresh B. Babu, Raymond Chung

**Affiliations:** 1 St George’s Hospital Medical School, Cranmer Terrace, Tooting, London, SW17 0RE UK; 2 Department of Diagnostic Radiology, Khoo Teck Puat Hospital, 90 Yishun Central, Singapore, 768828 Singapore

**Keywords:** BRTO, Ectopic Varices, Colonic Varices, Liver cirrhosis

## Abstract

**Background:**

Ectopic varices are uncommon and typically due to underlying liver cirrhosis. They can be located in the duodenum, small intestines, colon or rectum, and may result in massive haemorrhage. While established guidelines exist for the management of oesophageal and gastric variceal bleeding, this is currently lacking for colonic varices.

Beta-blockers, transjugular intrahepatic portosystemic shunt insertion and subtotal colectomy have been reported as management methods. However, there are only two other cases that have reported successfully treating colonic varices using balloon-occluded retrograde transvenous obliteration (BRTO), an endovascular procedure typically performed for gastric varices.

**Case presentation:**

A 55-year-old man with background of alcoholic liver cirrhosis presented with per-rectal bleeding due to caecal varices. Grade 2–3 oesophageal varices were identified on oesophago-gastro-duodenoscopy, and computed tomography showed multiple right para-colic portosystemic collaterals around the hepatic flexure and ascending colon. Colonoscopy confirmed fresh blood in the colon up to the caecum, with a submucosal varix deemed the most likely source of haemorrhage.

As transjugular intrahepatic portosystemic shunt insertion was potentially technically difficult, due to left portal vein thrombosis and a small right portal venous system, he underwent BRTO, which successfully embolised and thrombosed the colonic varices without complications.

**Conclusions:**

Whilst further studies are required to conclude its effectiveness and efficacy, BRTO may be considered a viable solution in managing ectopic, colonic, variceal haemorrhage especially when traditional techniques are unsuccessful or contraindicated.

## Background

Ectopic varices are portosystemic venous collaterals that result from portal hypertension occurring in any location other than the oesophageal or gastric region (Norton et al. 1998). They are uncommon but may result in massive haemorrhage and typically due to underlying liver cirrhosis. A Japanese nationwide survey revealed only 173 cases over a five-year period, from 2001 to 2005, of which there were 57 duodenal, 11 small intestinal, 77 rectal and only 6 cases of colonic varices (Watanabe et al. 2010).

Although there are established guidelines for the management of oesophageal and gastric variceal bleeding, this is currently lacking for colonic varices. Various treatment methods have been reported, including the use of beta-blockers, transjugular intrahepatic portosystemic shunt (TIPS), and subtotal colectomy (El-Masry and Hu 2010; Francois et al. 2007; Haddad and Lacey 2014; Klein et al. 2003; Mehta et al. 2019; Mikat 1971; Shaper et al. 1996; Krishna et al. 2010; Langemets and Ilves 2017). However, management is typically dependent on local expertise, the underlying cause and the site of the varices.

We describe a case of caecal variceal bleeding in a patient with liver cirrhosis who presented with per-rectal bleeding. This was treated with balloon-occluded retrograde transvenous obliteration (BRTO), an endovascular procedure typically performed for gastric varices. BRTO involves the blockage of dilated outflow veins with a balloon, followed by injection of a sclerosing agent directly into the varix (Sabri and Saad 2011).

## Case presentation

A 55-year-old man with a background of alcoholic liver cirrhosis (Child-Pugh Class B) presented with a two-day history of fresh per-rectal bleeding and postural dizziness. Whilst he had a history of vomiting, he did not have hematemesis. As a known patient with oesophageal varices, he was on oral propranolol, 10 mg twice daily.

On examination, the patient was jaundiced with conjunctival pallor. Clubbing was present and a fine tremor was observed in both hands. His abdomen was soft with mild tenderness, and a per-rectal examination revealed fresh blood.

At the time of admission, the patient was alert and orientated, haemodynamically stable with a blood pressure of 135/98 mmHg, borderline tachycardic with a heart rate of 101 bpm, had oxygen saturations of 100% on air, and a temperature of 36.7 °C. Laboratory tests revealed haemoglobin 6.0 g/dl, platelets 134, albumin 25, bilirubin 50, ALP 91, ALT 59, AST 104, GGT 121, pro-thrombin time 10.8 and INR 1.03.

The patient was transferred to the intensive care unit where he was intubated, and oesophago-gastro-duodenoscopy was subsequently performed. This revealed four columns of grade 2–3 oesophageal varices with red wale signs (i.e. longitudinal red streaks on the varices), portal hypertensive gastropathy and a small duodenal ulcer, but no active bleeding. As the patient had fresh per-rectal bleeding of unknown aetiology, he was referred for computed tomography (CT).

**Fig. 1:**
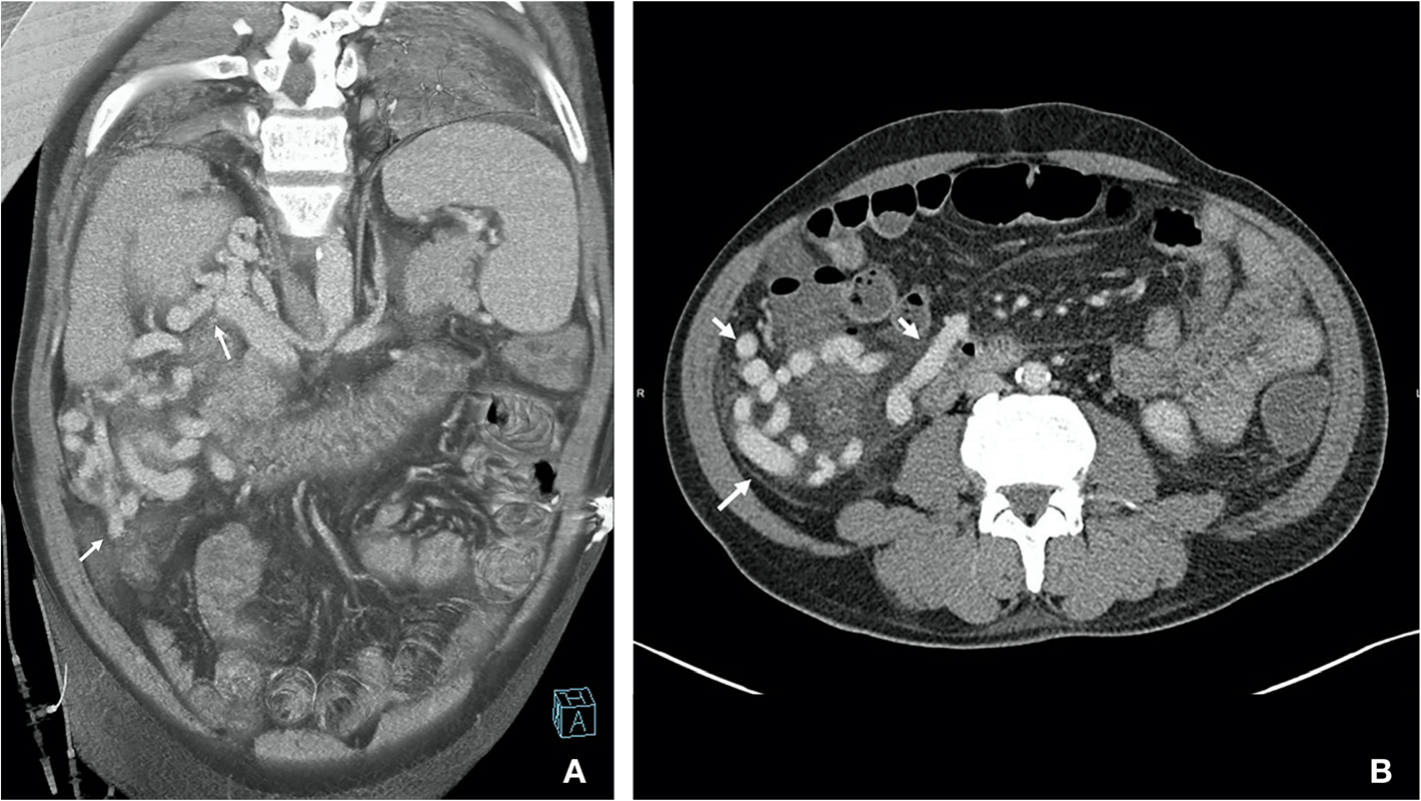
**a** Coronal thin MIP image from portal venous phase showing pericolic varices (2 white arrows) with dilated right renal vein. **b** Axial CT image in portal venous phase showing large peri-colic collaterals around the ascending colon (3 white arrows)

Triphasic (non-contrast, arterial and porto-venous phases post 85mls of Omnipaque 350 mg/ml delivered at a rate of 4 ml/s with bolus tracking performed on a Siemens Somatom Diefinition Flash) CT showed multiple right para-colic portosystemic collaterals around the hepatic flexure and ascending colon (Fig. 1), in addition to the known cirrhosis and features of portal hypertension. No active extravasation was noted, and the patient was given a somatostatin infusion, 500 mcg/h, before colonoscopy was performed. Colonoscopy confirmed fresh blood in the colon up to the caecum, with a submucosal varix deemed the most likely source of haemorrhage. Endoscopic clips were placed adjacent to the varix to act as markers to guide subsequent therapy.

The patient was administered intravenous vasopressin (Terlipressin 2 mg 4-hourly), a beta blocker (Carvedilol titrated up to 18.75 mg twice daily) and multiple units of platelets, packed cells and fresh frozen plasma. His haemoglobin improved to 8.4 g/dl and he remained haemodynamically stable for the next few days. After multi-disciplinary team discussion, he underwent balloon-occluded retrograde transvenous obliteration as TIPS was potentially technically difficult due to left portal vein thrombosis and a small right portal venous system.

### Balloon-occluded retrograde transverse obliteration (BRTO)

**Fig. 2:**
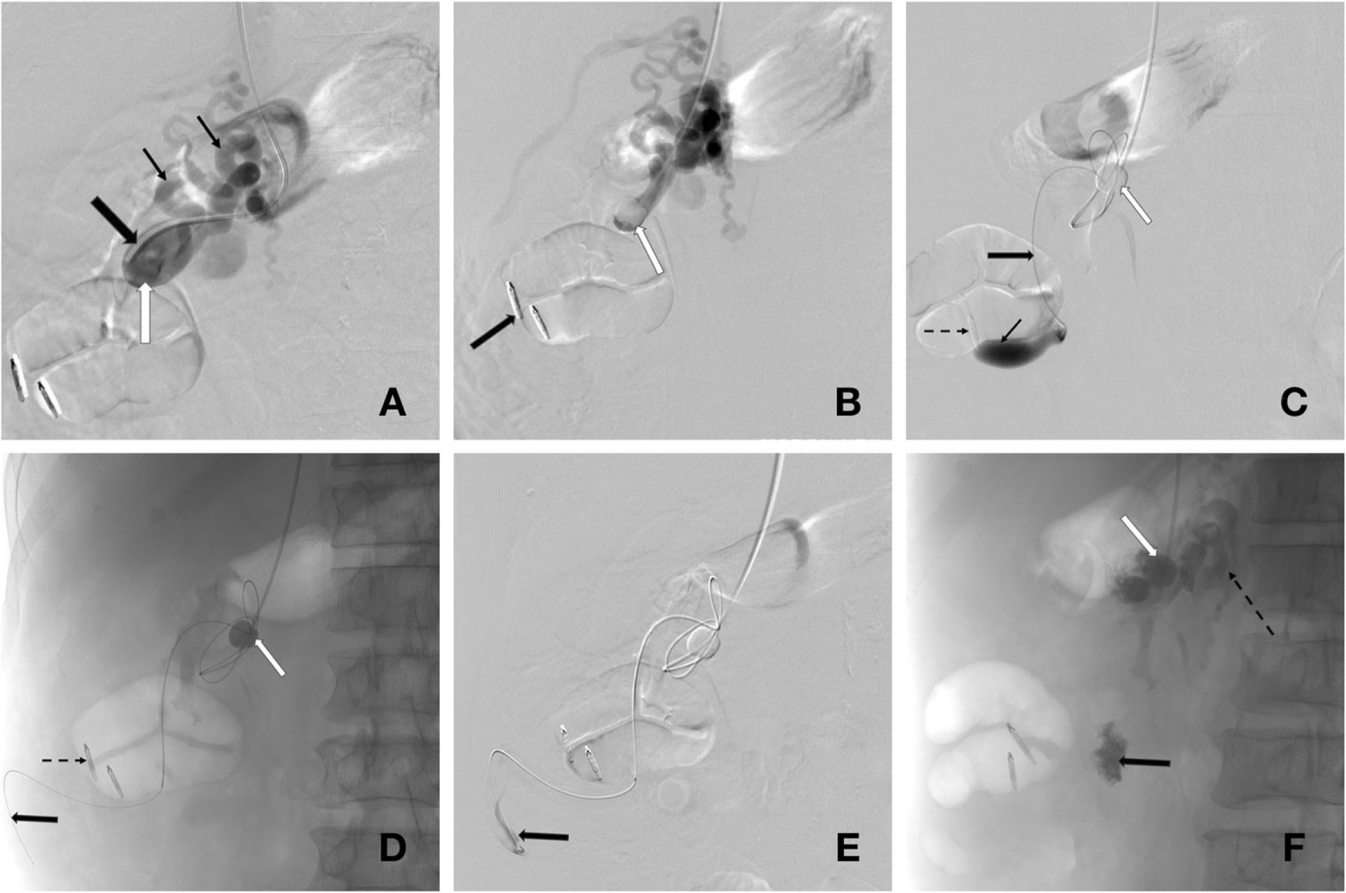
BRTO. **a** Reverse curve catheter (large black arrow) cannulating the right renal vein from right internal jugular venous access contrast opacifying the renal venous shunt (white arrow) and subsequent multiple tortuous porto-systemic collaterals (small black arrows). **b** Reverse curve catheter has been exchanged for a balloon occlusion catheter which has been inflated (white arrow) in the shunt. Two endoscopic clips denote the location of the culprit varix (black arrow). **c** Microcatheter (large black arrow), inserted through the central lumen of the inflated balloon occlusion catheter (white arrow), cannulating the extensive colonic varices and opacifying the culprit varix (small black arrow) close to the endoscopic clips (black dashed arrow). **d** Fluoroscopic image depicting the inflated balloon occlusion catheter (white arrow), microcatheter (large black arrow) inserted deeper into the colonic varix beyond the level of the endoscopic clips (black dashed arrow). **e** Digital subtraction venography via the microcatheter confirming intraluminal colonic varix opacification (black arrow) prior to injection of sclerosant. **f** Following sclerotherapy and glue embolization (black arrow), the microcatheter has been removed. Fluoroscopic venogram via the balloon occlusion catheter (white arrow) post treatment with small retroperitoneal collateral filling (black dashed arrow) but no further filling of the culprit varix

The procedure was performed under general anaesthesia in the Interventional Radiology angiography suite. The right internal jugular vein was accessed, through which free and wedged hepatic venous pressures were measured with a calculated hepatic venous pressure gradient of 15 mmHg. The decision for BRTO was re-affirmed, considering his overall morbidities. Right renal venogram was performed with a 4Fr catheter confirming the large colo-renal shunt with multiple tortuous pericolic varices around the caecum and ascending colon. The large colo-renal shunt was catheterised via the right renal vein using a reverse curve catheter. A stiff wire was advanced deep into the tortuous shunt and the catheter was exchanged for an occlusion balloon catheter (6Fr, 8.5–11.5 mm) (Berentstein, Boston Scientific, Natick, MA, USA). Balloon occlusion venography (Fig. 2), in which a balloon catheter is inflated to occlude the venous shunt and contrast subsequently injected upstream/retrogradely, was performed to delineate the anatomy of the pericolic porto-systemic collaterals and drainage pattern. With the balloon inflated to achieve relative stasis of blood and prevent efflux of sclerosant, a microcatheter was advanced through the occlusion balloon catheter deep into the varix before injecting 3% sodium tetradecyl sulphate (STS) sclerosant with the aim of filling the entire varix. The occlusive balloon remained in situ for approximately 4 h until there was satisfactory stasis of sclerosant. Small residual variceal collaterals were embolised with N-Butyl cyanoacrylate (NBCA) glue. Venograms confirmed satisfactory embolisation of the abnormal pericolic varices. The balloon catheter was then deflated and removed.

**Fig. 3:**
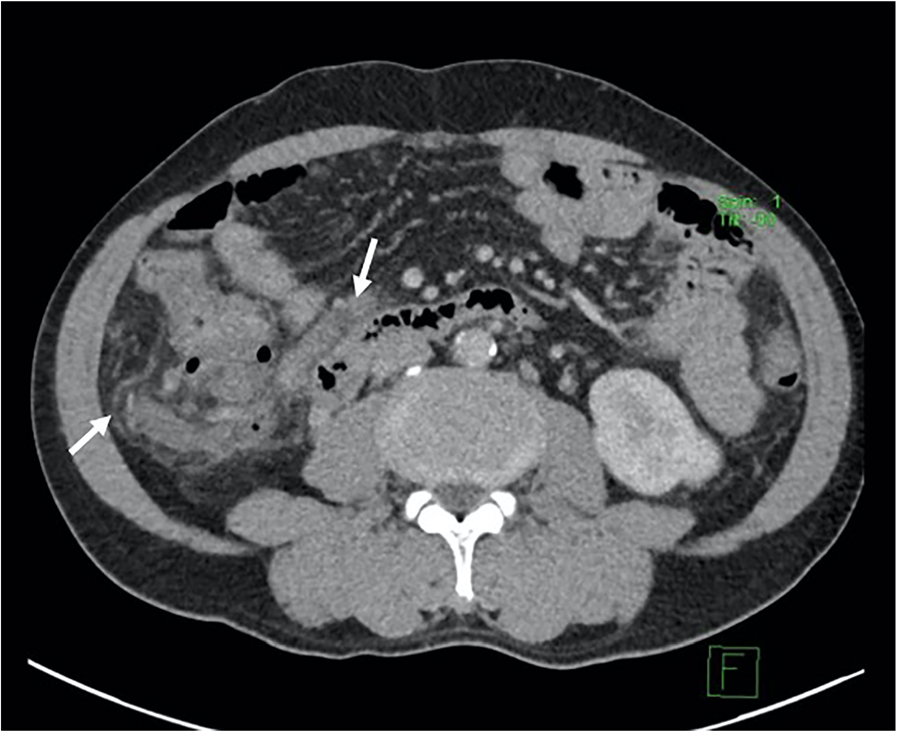
Follow-up computed tomography post-BRTO 2 months later in portal venous phase showing absence of variceal opacification compatible with variceal occlusion (white arrows). There was no evidence of colonic ischaemia

The patient remained hemodynamically stable following the procedure and experienced an uneventful post-procedure recovery. His Hb levels remained stable at 8.7 g/dl and there were no further episodes of gastrointestinal bleeding. An outpatient follow-up bi-phasic (portovenous and delayed post contrast with 70mls of Omnipaque 350 mg/ml) CT 2 months later confirmed thrombosed varices (Fig. 3).

## Discussion

Current UK guidelines from the British Society of Gastroenterology advise administering antibiotics, vasoconstrictors (e.g. Terlipressin) and variceal band ligation to control variceal haemorrhage in cirrhotic patients. In the event of satisfactory haemostasis, early TIPS (< 72 h after the index variceal bleed) is recommended in patients with Child-Pugh B cirrhosis and active bleeding or Child-Pugh C cirrhosis with Child’s score < 14 (Tripathi et al. 2015). Whilst BRTO is mentioned under ‘other radiological procedures’ that can be performed in the acute management of gastric variceal bleeding as an alternative to the more familiar therapeutic arms of non-selective beta blockers, endoscopy, TIPS, and surgery; it is rarely performed outside Asian centres (Saad 2011).

There are relatively few cases of ectopic varices treated with BRTO in current literature, including only 11 out of 168 (6.5%) cases in a five-year Japanese nationwide survey. These comprised of 7 duodenal, 2 small intestinal, 1 colonic and 1 stomal cases. Management of ectopic varices was most common by observation (41.1%) and endoscopic methods of injection sclerotherapy and variceal ligation (29.2%), with 7.7% requiring surgery (Watanabe et al. 2010). To the authors’ knowledge, there are three reported cases of attempted BRTO for colonic varices, of which two were successful (Anan et al. 2006; Matsumoto et al. 2018; Ko et al. 2013).

Anan et al. (Anan et al. 2006) described the first successful BRTO of descending colonic varices, thereby obliterating the portosystemic shunt, for the treatment of hepatic encephalopathy. Matsumoto et al. (Matsumoto et al. 2018) reported the second successful case, obliterating ascending colonic varices through the right testicular vein, preventing variceal rupture. Ko et al. (Ko et al. 2013) reported a failed BRTO attempt in a 38-year-old female with variceal bleeding of the ascending colon, which was eventually treated by venous coil embolisation and histoacryl injection.

As current literature is predominantly limited to case reports, there is little data available to develop guidelines on the role BRTO has in the management of ectopic, particularly, colonic, varices. Application of this technique is therefore extrapolated from the published evidence of BRTO in treating gastric variceal bleeds for which it is considered a good alternative to TIPS, effectively achieving haemostasis, as well as improving hepatic encephalopathy and liver function (Choi et al. 2003; Miyamoto et al. 2003). It is indicated for impending, prior or active variceal bleeding and in refractory debilitating hepatic encephalopathy. Relative contraindications include severe uncorrected coagulopathy, splenic vein thrombosis, portal vein thrombosis and uncontrolled oesophageal variceal bleeding (Saad et al. 2013).

In the setting of gastric varices, BRTO has been reported to achieve comparable or even better results than TIPS in terms of immediate bleeding control (96.2% vs 84.2%), rebleeding-free survival rate (38.6% vs 23.4%, adjusted HR 0.34, *p* = 0.001) and overall survival rates at 5 years (38.5% vs 34.4%, adjusted HR 0.44, *p* = 0.01) (Gimm et al. 2018). Complete obliteration of gastric fundal varices has been achieved in as high as 91% of cases, with successful complete obliteration translating to rebleed-free survival, and none of the patients experiencing a worsening of their Child-Pugh score (Hiraga et al. 2007). It also fares favourably, with significantly lower rates of rebleeding one-year post-procedure, when compared to endoscopic cyanoacrylate intra-varix injection (3.5% vs 22.0%) (Stein et al. 2019).

Whilst there are no studies that have investigated the long-term effects of BRTO for colonic varices, Akahoshi et al. (Akahoshi et al. 2008) reported a cumulative rebleeding rate of 2.4%, 2.4% and 14.3%, and cumulative survival rate of 96.5%, 81.7% and 79.0% at 3, 5 and 8 years, respectively, for gastric varices treated with BRTO. They also reported that none of the patient mortalities were attributed to variceal bleeding. However, Cho et al. (Cho et al. 2007) reported that 67.7% of patients who underwent BRTO for gastric varices developed new oesophageal varices, red spots on pre-existing oesophageal varices or oesophageal variceal bleeding. This occurred at a mean of 539 days, with 16.1% also developing gastric cardiac varices.

## Conclusion

Whilst further studies are required to conclude its effectiveness and efficacy, BRTO may be considered a viable solution in managing ectopic, colonic, variceal haemorrhage especially when traditional techniques are unsuccessful or contraindicated.

## Data Availability

Not applicable.

## Abbreviations

ALP: Alkaline phosphatase
ALT: Alanine transaminase
AST: Aspartate aminotransferase
BRTO: Balloon-occluded retrograde transvenous obliteration
CT: Computed tomography
GGT: Gamma-glutamyltransferase
INR: International normalised ratio
NBCA: N-butyl cyanoacrylate
STS: Sodium tetradecyl sulphate
